# Associations Between Handgrip Strength and Dementia Risk, Cognition, and Neuroimaging Outcomes in the UK Biobank Cohort Study

**DOI:** 10.1001/jamanetworkopen.2022.18314

**Published:** 2022-06-23

**Authors:** Kate A. Duchowny, Sarah F. Ackley, Willa D. Brenowitz, Jingxuan Wang, Scott C. Zimmerman, Michelle R. Caunca, M. Maria Glymour

**Affiliations:** 1 Department of Epidemiology and Biostatistics, University of California, San Francisco; 2 Department of Psychiatry and Behavioral Sciences, University of California, San Francisco; 3 Department of Neurology, University of California, San Francisco

## Abstract

**Importance:**

The associations between muscle strength and cognitive outcomes have sparked interest in interventions that increase muscle strength for prevention of dementia, but the associations between muscle strength and cognitive aging are unclear, particularly among middle-aged adults.

**Objective:**

To evaluate the association between handgrip strength (HGS) and dementia, reduced cognition, and poorer neuroimaging outcomes in a UK population of middle-aged adults.

**Design, Setting, and Participants:**

This cohort study evaluated UK Biobank participants aged 39 to 73 years enrolled from 2006 to 2010 with measured HGS and prospectively followed up for dementia diagnosis. Data were analyzed from October 2021 to April 2022.

**Exposures:**

HGS assessed in both hands via dynamometer.

**Main Outcomes and Measures:**

Outcomes included cognitive test scores (fluid intelligence and prospective memory), brain magnetic resonance imaging measures (total brain volume, white matter hyperintensity, and hippocampal volume), and incident dementia (all-cause, vascular, and Alzheimer disease [AD] from primary care, hospital, or death records) over a median (IQR) of 11.7 (11.0-12.4) years of follow-up. Mixed-effects linear and logistic regressions and Cox proportional-hazard models were used to estimate associations, stratified by gender and adjusted for covariates. Estimates are presented per 5-kg decrement in HGS. To evaluate reverse causation, we assessed whether a polygenic risk score for AD is associated with HGS.

**Results:**

A subsample of 190 406 adult participants in the UK Biobank (mean [SD] age, 56.5 [8.1] years; 102 735 women [54%]) were evaluated. A 5-kg decrement in HGS was associated with lower fluid intelligence scores in men (β, –0.007; 95% CI, –0.010 to –0.003) and women (β, –0.04; 95% CI, –0.05 to –0.04. A 5-kg decrement in HGS was associated with worse odds of correctly responding to a prospective memory task for men (odds ratio, 0.91; 95% CI, 0.90 to 0.92) and women (odds ratio, 0.88; 95% CI, 0.87 to 0.90). A 5-kg decrement in HGS was associated with greater white matter hyperintensity volume in men (β, 92.22; 95% CI, 31.09 to 153.35) and women (β, 83.56; 95% CI, 13.54 to 153.58). A 5-kg decrement in HGS was associated with incident dementia for men (hazard ratio, 1.20; 95% CI, 1.12 to 1.28) and women (hazard ratio, 1.12; 95% CI, 1.00 to 1.26). The AD genetic risk score was not significantly associated with HGS.

**Conclusions and Relevance:**

These findings suggest that HGS is associated with measures of neurocognitive brain health among men and women and they add to a growing body of research indicating that interventions designed to increase muscle strength, particularly among middle-aged adults, may hold promise for the maintenance of neurocognitive brain health.

## Key Points

**Question:**

Is reduced muscle strength, as measured by handgrip strength, associated with higher risk of dementia, poorer neuroimaging outcomes, and reduced cognition in both men and women?

**Findings:**

This cohort study of 190 406 adults in the United Kingdom found associations for both men and women across multiple outcomes and with multiple adjustment strategies. Handgrip strength was associated with fluid intelligence, prospective memory, and dementia diagnoses; this association was most pronounced for vascular dementia.

**Meaning:**

These findings add to a growing body of research suggesting that interventions designed to increase muscle strength, particularly among middle-aged adults, may hold promise for the maintenance of neurocognitive brain health.

## Teaser

This cohort study evaluates the association between handgrip strength and dementia, reduced cognition, and poorer neuroimaging outcomes in a UK population of middle-aged adults.

## Introduction

Muscle strength^1^ is associated with numerous health outcomes, including measures of cognitive aging.^2,3,4,5,6,7^ This association suggests there may be potential benefits of strength training for delay of age-related cognitive loss or dementia, but there is a dearth of evidence to inform such interventions. For example, the most relevant outcomes (cognitive function, clinical dementia, or neuroimaging), potential mechanisms, variability in outcomes across gender or age groups, and possible spurious explanations for the association have not been evaluated. Handgrip strength (HGS) is a reliable measure of muscle strength feasible to evaluate in large samples. HGS offers the opportunity for a closer examination of how muscle strength is associated with multiple neurocognitive outcomes.

Numerous studies^8,9^ show that HGS is associated with worse cognitive test scores, but these associations are vulnerable to reverse causation bias if incipient dementia influences HGS. A recent systematic review^10^ identified 10 studies of HGS and incident dementia, but only 2 used clinically recorded dementia outcomes,^11,12^ and none included sufficient sample size or diversity to evaluate gender or age differences in the associations. Only 2 prior studies^13,14^ have evaluated HGS and neuroimaging measures, and sample sizes in both studies were too small to provide precise gender-stratified estimates.

Almost no evidence is available on whether the association between HGS and neurocognitive outcomes differs between midlife and late life. Midlife is a particularly important window; midlife precedes onset of nearly all dementia, and interventions in midlife have the largest benefit to muscle strength.^15,16^ Furthermore, evaluating associations in midlife can help rule out reverse causation from dementia to HGS, because symptomatic dementia is very rare before age 65 years. Examining gender-stratified associations is also important since there may be significant differences in effect sizes due to differences in the distribution of HGS by gender. Additionally, some studies conclude that age-specific incidence of dementia may be higher for older women,^17^ and prior studies^18,19^ have observed reduced muscle strength among postmenopausal women.

In this study, we leveraged data from the UK Biobank, a large, well-characterized sample of middle-aged and older men and women who were dementia free at baseline to examine the associations of HGS with incident dementia, neuroimaging correlates, and cognition. We augmented findings on the association of HGS with cognition by conducting a mendelian randomization analysis of the association between genetic risk of dementia and HGS. We hypothesized that reduced muscle strength, as measured by HGS, would be associated with higher risk of dementia, poorer neuroimaging outcomes, and reduced cognition in both men and women. We also hypothesized that genetic risk of dementia would not be associated with HGS.

## Methods

### Study Population and Sample

Ethical approval was obtained from the National Health Service National Research Ethics Service. All participants provided written informed consent. This study follows the Strengthening the Reporting of Observational Studies in Epidemiology (STROBE) reporting guideline.

A total of 502 490 UK Biobank (UKB) participants aged 39 to 73 years without dementia were enrolled during 2006 to 2010. Cognition and dementia diagnoses were obtained from a combination of neuropsychological assessments and records from primary care and hospital settings, which were available for a subset of participants.^20^ The analytical samples for each analysis varied according to the number of participants with available data. Analytical samples range from approximately 40 000 participants for neuroimaging outcomes to approximately 200 000 participants for dementia incidence. Individuals with dementia diagnosed before the age of 40 years were excluded from all analyses. Field identifications used in this analysis are described in eTable 1 in the Supplement.

### Primary Exposure

HGS was measured by a trained research staff member using a Jamar J00105 hydraulic hand dynamometer (Jamar) following standardized procedures.^20,21^ Briefly, participants sat upright keeping their elbow close to their torso and forearm positioned on an armrest with the thumb facing upward. Participants were asked to squeeze the handle of the dynamometer as strongly as they could for approximately 3 seconds. Each participant contributed a right and left HGS measurement in kilograms at each clinic visit. We obtained 1 measure from the left hand and 1 from the right hand. The maximum score from both hands was then included. Participants had up to 4 study visits at which HGS was measured.

### Primary Outcomes

Incident dementia was ascertained using diagnoses obtained from primary care, hospital inpatient, and death registry records. Only individuals with a confirmed primary care linkage were included in analyses of incident dementia. We used the date of diagnosis according to the earliest dementia code recorded, irrespective of the source used. Similar merging with primary care records was performed for Alzheimer disease (AD) and vascular dementia diagnoses. Primary care records were queried with *International Classification of Diseases, Ninth Revision* and *International Statistical Classification of Diseases and Related Health Problems, Tenth Revision* for dementia, AD, and vascular dementia (eTable 2 in the Supplement). Participant time in years was calculated from the date of HGS assessment until the date of dementia diagnosis, date of loss to follow-up, date of death, censoring (December 4, 2020), or updating date of HGS.

### Cognitive Outcomes

Fluid intelligence, a 13-item task of problem-solving requiring logic and reasoning ability, was evaluated using a touch screen at UKB assessment center visits.^20^ This measure was available for all participants. Prospective memory, a type of episodic memory, measures an individual's memory for future tasks and was assessed via touch screen with a single instruction to be recalled later in the session. Values were coded as 1 for correct on the first attempt, and 0 otherwise.

### Neuroimaging Outcomes

We used global and regional brain imaging derived phenotypes provided by the UKB brain imaging team. Details on imaging acquisition and the imaging processing are available elsewhere.^20^ We examined total brain volume, hippocampal volume, and white matter hyperintensity (WMH) volume, which were ascertained using T1-weighted and T2-weighted fluid-attenuated inversion recovery volumes structural brain magnetic resonance imaging.^22^ Total brain volume and hippocampal volume were scaled by intracranial volume (divided by intracranial volume over mean intracranial volume across participants), whereas sensitivity analyses additionally examined associations with crude volumes. Additional information for all outcomes is provided in the eMethods in the Supplement.

### Covariates

**Figure 1.**
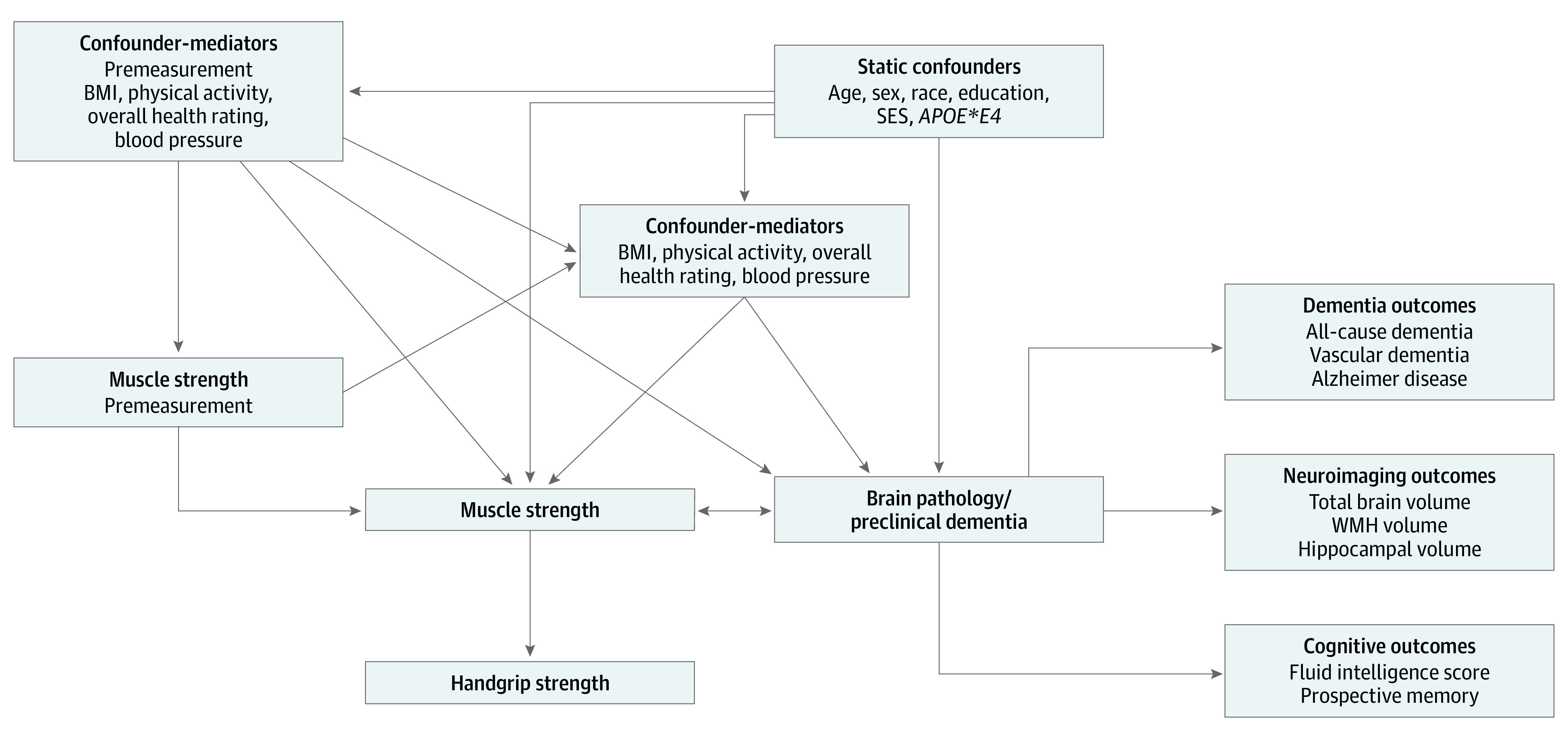
Conceptual Model Motivating Analyses of the Outcomes of Muscle Strength as Measured by Handgrip Strength, as Associated With Cognitive, Neuroimaging, and Dementia Outcomes Variables body mass index (BMI), physical activity, overall health rating, blood pressure, and preclinical dementia are theoretically relevant but were not measured in the UK Biobank data set. The base model (model 1) adjusts for only gender and age, whereas the confounder model (model 2) adjusts for all static confounders and the confounder-mediator model (model 3) additionally adjusts for all confounder mediators. Model outcomes include dementia outcomes (clinical diagnosis), neuroimaging outcomes, and cognitive outcomes. Outcomes were selected to provide as much insight as possible into potential mechanisms, for example implicating vascular, Alzheimer, or other disease processes. APOE*E4 indicates apolipoprotein E4; SES, socioeconomic status; WMH, white matter hyperintensity.

Figure 1 provides a conceptual model for covariate selection. The following covariates were included in our models: age and age squared (centered), education (dichotomized as high school equivalent; UK A levels), self-reported race (Asian, Black, White, and multiracial), assessment center, Townsend Deprivation Index at recruitment (an index measure based on a composite score derived from 4 key variables—unemployment, overcrowded household, non–car ownership, and non–home ownership index—with higher scores representing higher levels of deprivation),^23^ number of days per week of at least 10 minutes of moderate physical activity, clinic-measured body mass index (calculated as weight in kilograms divided by height in meters squared), overall health rating (response to the question, “In general how would you rate your overall health?”), and systolic blood pressure. Additional information on how the categories were collapsed for both race and education are provided in the eMethods in the Supplement. Race was included as a socially constructed variable that serves as a proxy in capturing variation in the lived experience across the life course. Of note, because there were too few dementia events in some higher education groups, we dichotomized education at the equivalent of high school completion, and used this categorization consistently across models.

### Timing of Measurement Ascertainment

Our analysis included those individuals who were dementia free at baseline. Thus, all HGS measures occurred before diagnosis and before the neuroimaging assessments. Dementia diagnoses were obtained from medical records and, therefore, were assessed independently of HGS. For the cognition models, HGS was assessed contemporaneously with the cognitive measures during the clinic visit.

### Statistical Analysis

#### Analytical Approach

The following modeling building strategy was used for each outcome. Given known gender differences in HGS and dementia risk across the life course, all models were stratified by gender.^24,25^ Model 1 (ie, the base model) included only age and age squared at time of HGS assessment. We adjusted for age squared to avoid residual confounding due to cognitive decline and dementia diagnoses accelerating with increasing age. Model 2 (ie, the confounder model) included all hypothesized confounders expected to stay constant through follow-up: assessment center, Townsend Deprivation Index at recruitment, race, education (dichotomized by high school attainment or equivalent), and number of apolipoprotein E4 (APOE*E4) alleles. The final model (model 3) added variables that could be considered both hypothesized confounders and mediators: body mass index, moderate physical activity (number of days per week of ≥10 minutes activity), overall health rating, and systolic blood pressure. We consider model 2 our primary results. Effect estimates from the final model (model 3) should be interpreted with caution as these results may represent overcontrol because they are conditioned on a confounder-mediator.^26^ Since dementia is typically diagnosed at age 65 years or older, models were stratified by midlife (<65 years) and older age adulthood (≥65 years) to examine differences by age. Information on missingness across the whole sample at the first visit is provided in eTable 3 in the Supplement.

Cox proportional hazard models were used to assess the relative hazard of dementia associated with lower HGS, using time since HGS assessment as the timescale and stratification by age at assessment.^27^ Individuals were followed for a mean (SD) of 11.4 (1.63) years, totaling 2 196 310 person-years of follow-up. For individuals with repeated measures, HGS was time updated. Death from any nondementia cause, as obtained from the death registry data, was considered a competing event. Proportional hazard assumptions were met for all-cause dementia models at a significance threshold of *P* < .05 (eMethods in the Supplement).^28^ To account for repeated and contemporaneous HGS and cognitive measures, we used mixed-effects linear and logistic models to examine the association between HGS and cognitive measures. To account for both repeated neuroimaging measures, we used mixed-effects linear models to examine the association between baseline HGS and magnetic resonance imaging volumetric measures. All mixed-effects models included a random intercept by individual and fixed slope while adjusting for age and age squared as orthogonal polynomials.^29^

#### AD Genetic-Risk Analysis

To investigate the potential for reverse causation and shared genetics between HGS and incipient dementia (see the conceptual model in Figure 1), we determined the association between HGS and number of APOE*E4 alleles and a polygenic risk score for Alzheimer dementia, described elsewhere and previously validated in UKB, in European ancestry individuals.^30^ Since genetic risk is determined at conception, these associations are adjusted for only the first 10 principal components and age and age-squared at HGS assessment. A significant association between HGS and genetic risk, as determined by the number of APOE*E4 alleles or the polygenic risk score, would suggest that HGS is affected by pathological changes in preclinical dementia or shared genetic pathways. This approach has been validated for other potential early symptoms of AD, including cognition and body mass index.^30,31^

Data analysis was conducted using R statistical software version 4.0.4 (R Project for Statistical Computing) from October 2021 to April 2022. All tests were 2-tailed.

## Results

**Figure 2.**
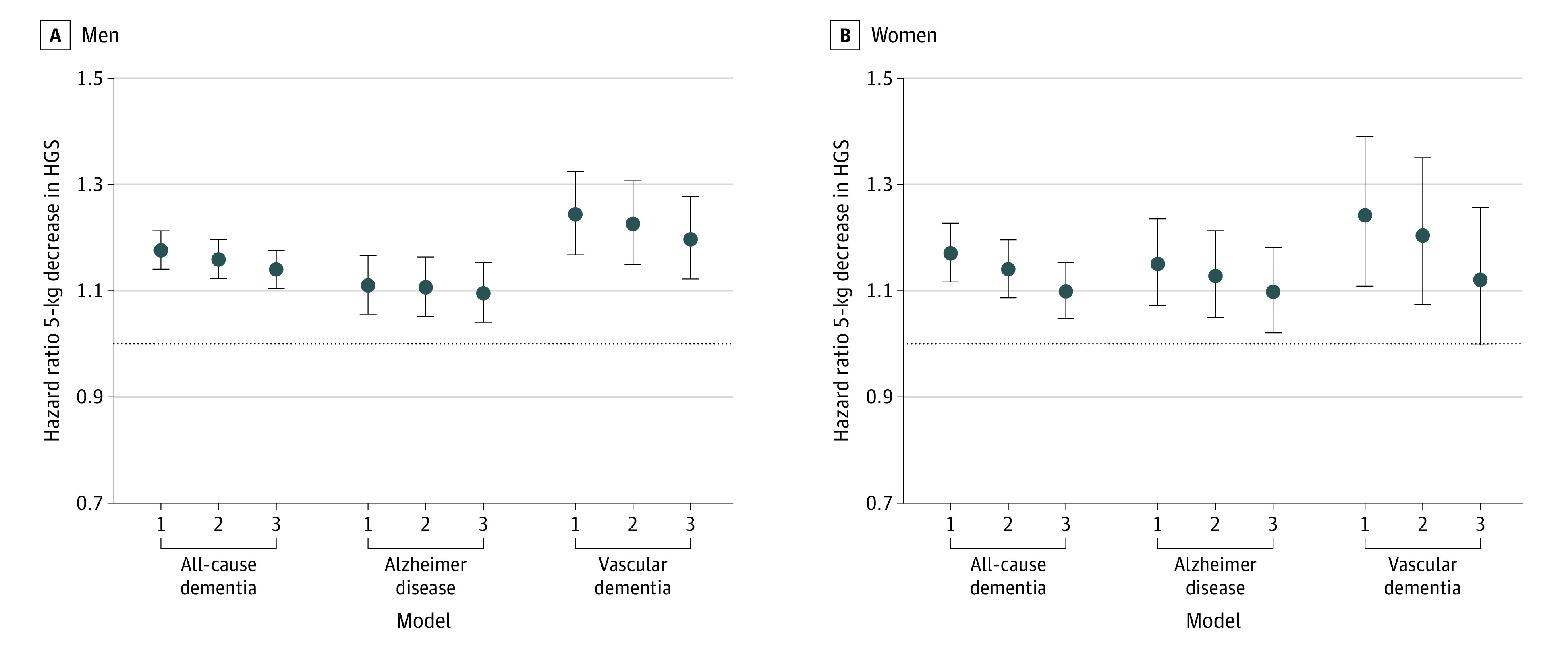
Gender-Stratified Hazard Ratios for Dementia, Alzheimer Disease, and Vascular Dementia Diagnoses Associated With a 5-kg Decrement in Handgrip Strength (HGS) Among 190 406 UK Biobank Participants Model 1 was adjusted for gender and age, model 2 was adjusted for all static confounders, and model 3 was additionally adjusted for baseline values of time-varying confounder-mediators. The null is shown as a horizontal dotted line.

**Figure 3.**
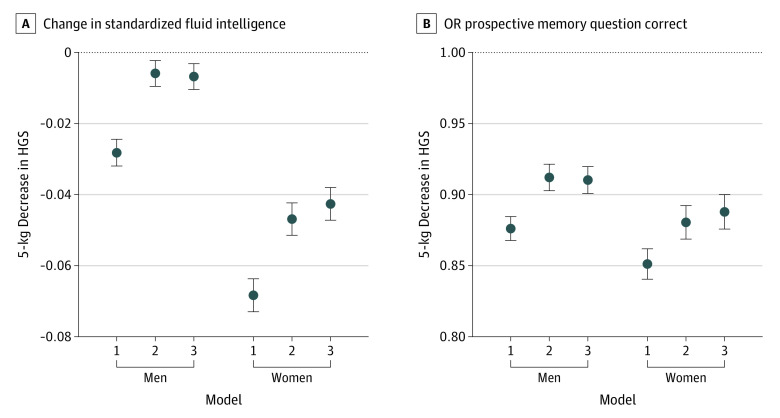
Gender-Stratified Linear Regression Estimates for the Association of a 5-kg Decrement in Handgrip Strength (HGS) With Fluid Intelligence Score and Correct Prospective Memory Response Among 153 397 UK Biobank Participants Model 1 was adjusted for gender and age, model 2 was adjusted for all static confounders, and model 3 was additionally adjusted for baseline values of time-varying confounder-mediators. The null is shown as a horizontal dotted line.

The main sample included 190 406 adults with a mean (SD) age of 56.5 (8.1) years; 102 735 (54%) were women and 182 073 (95.6%) were White (Table and eTable 4, eTable 5, and eTable 6 in the Supplement ). Main results are provided in Figure 2 and Figure 3. We also provide effect estimates for all models in eTable 7, eTable 8, eTable 9, eTable 10, eTable 11, eTable 12, eTable 13, eTable 14, and eTable 15 in the Supplement.

**Table.** Baseline Demographic Characteristics Among Individuals Enrolled in UK Biobank and Evaluated for Handgrip Strength and Dementia

Characteristic	Mean (SD)		
	Women (n = 102 735)	Men (n = 87 671)	Total (N = 190 406)
Age, y	56.27 (8.0)	56.71 (8.2)	56.47 (8.1)
High school equivalent or more schooling, participants, No. (%)	65 109 (63.4)	60 556 (69.1)	125 665 (66.0)
Race, participants, No. (%)			
Asian	2044 (2.0)	2224 (2.5)	4268 (2.2)
Black	1388 (1.4)	1001 (1.1)	2389 (1.3)
Multiracial	962 (0.9)	714 (0.8)	1676 (0.9)
White	98 341 (95.7)	83 732 (95.5)	182 073 (95.6)
Body mass index^a^	27.05 (5.1)	27.86 (4.2)	27.42 (4.7)
Townsend Deprivation Index	–1.46 (2.9)	–1.41 (3.0)	–1.44 (3.0)
Days per week of moderate physical activity	3.63 (2.3)	3.63 (2.3)	3.63 (2.3)
Systolic blood pressure, mm Hg	137.31 (20.2)	143.02 (18.5)	139.94 (19.6)
Overall health rating, participants, No. (%)			
Prefer not to answer	27 (0.0)	26 (0.0)	53 (0.0)
Do not know	327 (0.3)	279 (0.3)	606 (0.3)
Excellent	17 380 (16.9)	13 535 (15.4)	30 915 (16.2)
Good	61 680 (60.0)	49 566 (56.5)	111 246 (58.4)
Fair	19 501 (19.0)	19 930 (22.7)	39 431 (20.7)
Poor	3820 (3.7)	4335 (4.9)	8155 (4.3)
APOE*E4 carrier, participants, No. (%)	29 102 (28.3)	24 876 (28.4)	53 978 (28.3)
Baseline handgrip strength, kg	25.16 (6.3)	41.83 (9.0)	32.84 (11.3)

Abbreviation: APOE*E4, apolipoprotein E4.

^a^ Body mass index is calculated as weight in kilograms divided by height in meters squared.

### Dementia Outcomes

Figure 2 show results for the association of HGS and risk of dementia diagnosis. In men, the hazard of developing all-cause dementia was elevated by 16% (hazard ratio [HR], 1.16; 95% CI, 1.12-1.20) for every 5-kg decrease in HGS (model 2) and changed little with additional covariates. In women, every 5-kg decrease in HGS was similarly associated with a 14% increase in the hazard of all-cause dementia (model 2 HR, 1.14; 95% CI, 1.09-1.20). Lower HGS was associated with incident dementia for men (HR, 1.20; 95% CI, 1.12-1.28) and women (HR, 1.12; 95% CI, 1.00-1.26). To contextualize the HGS results, a 1-year increase in age is associated with a 16% increase in hazard of dementia for both men (HR, 1.16; 95% CI, 1.14-1.17) and women (HR, 1.16; 95% CI, 1.15-1.17). For both men and women, HGS was associated with incidence of both AD and vascular dementia, with slightly larger point estimates for vascular dementia (Figure 2). Kaplan-Meier curves are provided in eFigure 1 and eFigure 2 in the Supplement.

### Cognitive Outcomes

Estimates of the association between a 5-kg decrement in HGS and cognitive assessments in 153 397 men and women are shown in Figure 3 (see also eTable 4 in the Supplement). A 5-kg decrement in HGS was associated with slightly lower fluid intelligence in men (β, –0.007; 95% CI, –0.010 to –0.003) and women (β, –0.04, 95% CI, –0.05 to –0.04) (model 2). An association was observed in women (β, –0.068; 95% CI, –0.073 to –0.064) (model 2). Every 5-kg lower HGS was associated with a 9% lower odds of recalling the test item correctly among men (odds ratio [OR], 0.91; 95% CI, 0.90 to 0.92) and 12% among women (OR, 0.88; 95% CI, 0.87 to 0.89) (Figure 3, model 2).

### Neuroimaging Outcomes

**Figure 4.**
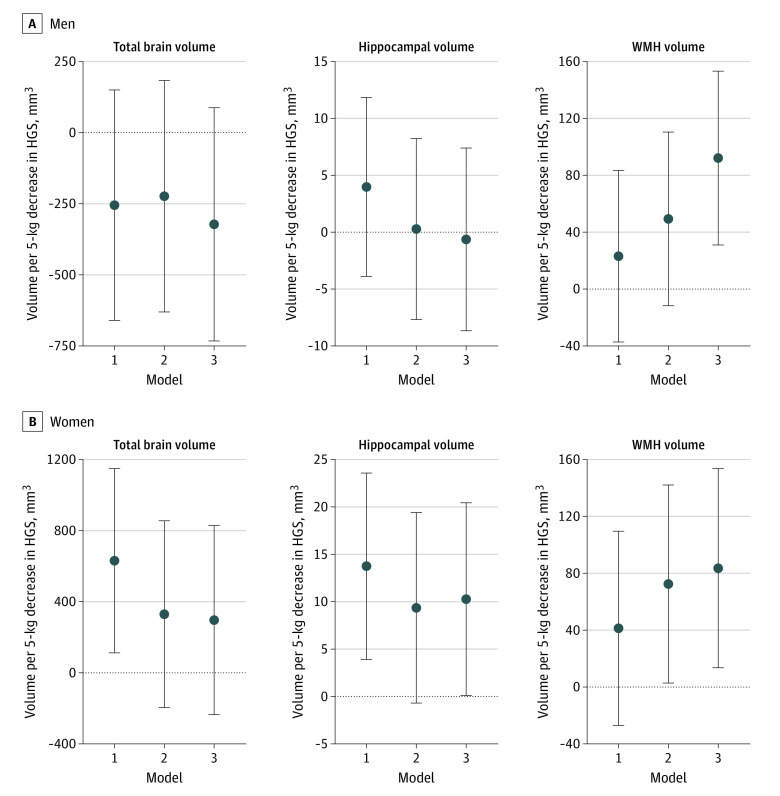
Gender-Stratified Linear Regression Estimates for the Association of a 5-kg Decrement in Handgrip Strength (HGS) With Total Brain Volume, Hippocampal Volume, and White Matter Hyperintensity (WMH) Volume Among 38 643 UK Biobank Participants Model 1 was adjusted for gender and age, model 2 was adjusted for all static confounders, and model 3 was additionally adjusted for baseline values of time-varying confounder-mediators. The null is shown as a horizontal dotted line.

Among the 38 643 men and women (eTable 5 in the Supplement), there was no association between HGS and total brain volume (Figure 4, model 3). HGS was not associated with hippocampal volume among men and was associated with hippocampal volume in women only in a fully adjusted model (β per 5-kg difference in HGS, 10.27, 95% CI, 0.10 to 20.43) (Figure 4, model 3). HGS was associated with WMH volume among both men and women. With adjustment for model 2 covariates, each 5-kg lower HGS was associated with 49.42 mm^3^ (95% CI, –11.67 to 110.51 mm^3^) WMH for men and 72.46 mm^3^ (95% CI, 2.78 to 142.14 mm^3^) for women. In contrast to all other outcomes, inclusion of additional covariates increased the magnitude and significance of coefficient estimates for WMH: in the fully adjusted model, every 5-kg lower HGS was associated with 92.22 mm^3^ (95% CI, 31.09 to 153.35 mm^3^) larger WHM volume in men and 83.56 mm^3^ (95% CI, 13.54 to 153.58 mm^3^) larger volume in women (Figure 4, model 3).

### Midlife vs Older Adult Neurocognitive Brain Health

Associations between HGS and neuroimaging and cognitive outcomes were similar in midlife (<65 years) and late life (65 years or older) (see eTable 10, eTable 11, eTable 12, eTable 13, and eTable 14 in the Supplement). Incident dementia diagnoses were not investigated since comparatively fewer diagnoses are made earlier in the life course.

### AD Genetic Risk Analysis

The AD polygenic risk score was not associated with HGS in models adjusted for age and age squared at HGS assessment and the first 10 genetic principal components in either men or women. Similarly, there was no association between number of APOE*E4 alleles and HGS among men or women younger than 65 years; for women aged 65 years and older, each additional APOE*E4 allele was associated with a slightly weaker HGS (β, –0.11; 95% CI, –0.20 to –0.02) (eFigure 3 and eTable 16 in the Supplement).

## Discussion

In this cohort of 190 406 individuals from a well-characterized, large, prospective study of adults in the United Kingdom, we examined the associations between HGS and multiple measures of neurocognitive brain health. We found associations for both men and women across multiple outcomes and with multiple adjustment strategies. Lower HGS was associated with decreased fluid intelligence, lower odds of a correct score on a prospective memory test, and increased dementia diagnoses. This association was most pronounced for vascular dementia. Lower HGS was associated with increased WMH volume for both men and women, but was not significantly associated with total brain volume or hippocampal volume. HGS is associated with cognition in midlife and late life and with WMH volume in midlife for both men and women. Genetic factors associated with risk for AD—variants known to already be exerting subtle cognitive effects in this sample^32^—had no or negligible association with HGS. In aggregate, these results suggest that even small changes in muscle strength might have a nontrivial association with vascular dementia risk.

Our results are consistent with studies that have found HGS and other measures of total body muscle strength are associated with cognitive status and incident dementia.^33,34^ We found that HGS was associated with both prospective memory and fluid intelligence, reliable proxies of overall cognitive status.^35,36^ Our findings corroborate prior cross-sectional^9^ and longitudinal findings,^37,38^ including a study by Firth et al^39^ in UKB focusing on HGS among individuals with schizophrenia, which reported higher HGS was associated with better task performance for visual memory, reaction time, number memory, and prospective memory. Our work builds on the findings of Firth et al^39^ by integrating results for cognitive outcomes with clinical dementia incidence and neuroimaging markers, alongside the evaluation of potential reverse causation using AD genetic factors associated with risk. In a systematic review of 15 longitudinal studies, Cui et al^10^ found that low HGS was associated with 2-fold higher risk of developing incident dementia. However, not all studies have uncovered a positive association between HGS and dementia.^11,12^ Our results extend these prior studies, which may have been underpowered, by examining dementia subtypes by gender and age in a larger sample.

Among men, the estimated association of HGS with vascular dementia was larger than the association of HGS with AD; this pattern was also observed among women, although the difference was smaller than among men and consistent with a chance finding. We also found that lower HGS was associated with greater WMH volume in both men and women. The larger observed associations between HGS and WMH volume than for total brain and hippocampal volume suggest that the association between HGS and dementia diagnosis may largely operate by vascular-related mechanisms. Prior research suggests an association between WMH volume and indicators of physical functioning with reduced muscle strength^40^ and gait speed^41^ associated with a greater WMH volume. Vascular endothelial dysfunction, characterized by decreased nitric oxide, increased inflammation, and oxidative stress, has been implicated in the pathogenesis of both vascular and AD^42,43^ and also in reduced muscle function, frailty and sarcopenia.^44,45^ Therefore, muscle strength may plausibly be associated with cognitive aging via these or related processes.

Previous studies did not evaluate the potential for reverse causation in associations between HGS and cognitive aging outcomes. We examined whether associations between HGS and cognition and HGS and neuroimaging markers were comparable in midlife and late life and found that HGS is associated with cognition in midlife and late life and WMH volume in midlife for both men and women. The magnitude of these associations in midlife may suggest that preclinical dementia-related pathology is not resulting in reduced muscle strength. In an additional analysis using a mendelian randomization framework,^30,31^ we found Alzheimer dementia genetic risk was not significantly associated with HGS, further indicating reverse causation is not driving the observed associations between HGS and cognitive, neuroimaging, and dementia outcomes. Although HGS and cognitive changes may be common results of an underlying aging process, we found that the genetic variants that are most associated with Alzheimer dementia are not associated with later changes in HGS, implying these are not dependent processes.

### Strengths and Limitations

There are several notable strengths to this study. The UKB Study is a large cohort of middle-aged and older adults with HGS, cognitive, and neuroimaging measures and linkages to medical records. The UKB also obtains clinical diagnoses from primary care, hospital inpatient, and death registry records, as well as self-report. The large sample size allowed us to explore differences by age and gender with sufficient power. To the best of our knowledge, this is the largest study to date that has simultaneously examined whether muscle strength is associated with multiple dementia subtypes, cognitive outcomes, and neuroimaging correlates. Although comparisons between men and women are challenging since it is unclear whether the same decrement change is equally relevant in estimating health outcomes, prior studies show improvements in muscle strength of 5 to 10 kg are feasible on a 2- to 3-month timescale in middle-aged adults^15,46^ Despite the fact that large differences are more difficult to achieve among older adults, improving muscle strength on the order of 5 kg is feasible over longer periods.^47,48,49^

However, despite these strengths, we note several limitations. This sample is highly selected with documented healthy volunteer bias,^50^ so generalizability to other populations may be limited. Additionally, we could only evaluate cross-sectional differences in HGS, which hindered the ability to evaluate change within an individual in relation to cognition. Furthermore, we did not adjust for multiple comparisons; however, we note that our outcomes are all correlated. We also emphasized only associations that were consistent in men and women and persisted with increasing adjustment.

## Conclusions

Our cohort study provides evidence that HGS is associated with several markers of cognitive aging, including neuroimaging markers of cerebral small vessel disease and subtypes of dementia. Our findings add to a small but growing body of research indicating that the association between muscle strength and dementia may be due to vascular mechanisms and that interventions designed to increase muscle strength, particularly among middle-aged adults, may hold promise for the maintenance of neurocognitive brain health.

## Notes

Supplement. **eTable 1.** Field IDs Used in Analyses **eTable 2.** Read Codes for Dementia Diagnoses **eMethods.** Primary Outcomes; Covariate Categorizations; Statistical Modeling Approach **eTable 3.** Missingness in First Visit for the Entire Sample **eTable 4.** Demographic Characteristics Among Individuals Completing Cognitive Testing **eTable 5.** Demographic Characteristics Among Individuals With Neuroimaging Measures **eTable 6.** Demographic Characteristics Among Individuals With GP Linkages, Stratified by Median HGS **eTable 7.** Gender-Stratified Linear Regression Coefficients for a 5-kg Decrement in Handgrip Strength and Fluid Intelligence and Prospective Memory **eTable 8.** Gender-Stratified Hazard Ratios for the Association Between Handgrip Strength and Incident All-Cause, Alzheimer, and Vascular Dementia **eTable 9.** Linear Regression Coefficients for the Association of a 5-kg Decrement in Handgrip Strength With Normalized and Nonnormalized Total Brain, Hippocampal, and White Matter Hyperintensity Volume, Stratified by Gender **eTable 10.** Age Less Than 65: Gender-Stratified Linear Regression Coefficients for a 5-kg Decrement in Handgrip Strength and Fluid Intelligence and Prospective Memory **eTable 11.** Age Less Than 65: Linear Regression Coefficients for the Association of a 5-kg Decrement in Handgrip Strength with Normalized and Nonnormalized Total Brain, Hippocampal, and White Matter Hyperintensity Volume, Stratified by Gender **eTable 12.** Age 65 and Older: Gender-Stratified Linear Regression Coefficients for a 5-kg Decrement in Handgrip Strength and Fluid Intelligence and Prospective Memory **eTable 13.** Age 65 and Older: Gender-Stratified Hazard Ratios for a 5-kg Decrement in Handgrip Strength and Incident All-Cause, Alzheimer, and Vascular Dementia **eTable 14.** Age 65 and Older: Linear Regression Coefficients for the Association of a 5-kg Decrement in Handgrip Strength With Normalized and Nonnormalized Total Brain, Hippocampal, and White Matter Hyperintensity Volume, Stratified by Gender **eTable 15.** Gender-Stratified Hazard Ratios for the Association Between Handgrip Strength and Incident All-Cause Dementia Including Whether Job Involves Manual Labor as a Confounder in Models 2 and 3 **eFigure 1.** Example Kaplan-Meier Curves for All-Cause Dementia for the 5th Age-Decile in Men **eFigure 2.** Example Kaplan-Meier Curves for All-Cause Dementia for the 5th Age-Decile in Women **eFigure 3.** Reverse Causation Schematic **eTable 16.** Associations Between Polygenetic Risk Score (PGS), *APOE*E4* Alleles and Handgrip Strength Stratified by Gender and Age
