# Supplementary material for: Associations Between Handgrip Strength and Dementia Risk, Cognition, and Neuroimaging Outcomes in the UK Biobank Cohort Study
Source: JAMA Netw Open. 2022 Jun 23;5(6):e2218314. doi: 10.1001/jamanetworkopen.2022.18314 (PMC9227006; doi:10.1001/jamanetworkopen.2022.18314)
Supplement: Supplement. — eTable 1. Field IDs Used in Analyses eTable 2. Read Codes for Dementia Diagnoses eMethods. Primary Outcomes; Covariate Categorizations; Statistical Modeling Approach eTable 3. Missingness in First Visit for the Entire Sample eTable 4. Demographic Characteristics Among Individuals Completing Cognitive Testing eTable 5. Demographic Characteristics Among Individuals With Neuroimaging Measures eTable 6. Demographic Characteristics Among Individuals With GP Linkages, Stratified by Median HGS eTable 7. Gender-Stratified Linear Regression Coefficients for a 5-kg Decrement in Handgrip Strength and Fluid Intelligence and Prospective Memory eTable 8. Gender-Stratified Hazard Ratios for the Association Between Handgrip Strength and Incident All-Cause, Alzheimer, and Vascular Dementia eTable 9. Linear Regression Coefficients for the Association of a 5-kg Decrement in Handgrip Strength With Normalized and Nonnormalized Total Brain, Hippocampal, and White Matter Hyperintensity Volume, Stratified by Gender eTable 10. Age Less Than 65: Gender-Stratified Linear Regression Coefficients for a 5-kg Decrement in Handgrip Strength and Fluid Intelligence and Prospective Memory eTable 11. Age Less Than 65: Linear Regression Coefficients for the Association of a 5-kg Decrement in Handgrip Strength with Normalized and Nonnormalized Total Brain, Hippocampal, and White Matter Hyperintensity Volume, Stratified by Gender eTable 12. Age 65 and Older: Gender-Stratified Linear Regression Coefficients for a 5-kg Decrement in Handgrip Strength and Fluid Intelligence and Prospective Memory eTable 13. Age 65 and Older: Gender-Stratified Hazard Ratios for a 5-kg Decrement in Handgrip Strength and Incident All-Cause, Alzheimer, and Vascular Dementia eTable 14. Age 65 and Older: Linear Regression Coefficients for the Association of a 5-kg Decrement in Handgrip Strength With Normalized and Nonnormalized Total Brain, Hippocampal, and White Matter Hyperintensity Volume, Stratified by Gender eTable 15. Gender [file jamanetwopen-e2218314-s001.pdf]

Supplemental Online Content

Duchowny KA, Ackley SF, Brenowitz WD, et al. Associations between handgrip strength and dementia risk, cognition, and neuroimaging outcomes in the UK Biobank cohort study. *JAMA Netw Open*. 2022;5(6):e2218314.
doi:10.1001/jamanetworkopen.2022.18314

eTable 1. Field IDs Used in Analyses
eTable 2. Read Codes for Dementia Diagnoses
eMethods. Primary Outcomes; Covariate Categorizations; Statistical Modeling Approach
eTable 3. Missingness in First Visit for the Entire Sample
eTable 4. Demographic Characteristics Among Individuals Completing Cognitive Testing
eTable 5. Demographic Characteristics Among Individuals With Neuroimaging Measures
eTable 6. Demographic Characteristics Among Individuals With GP Linkages, Stratified by Median HGS
eTable 7. Gender-Stratified Linear Regression Coefficients for a 5-kg Decrement in Handgrip Strength and Fluid Intelligence and Prospective Memory
eTable 8. Gender-Stratified Hazard Ratios for the Association Between Handgrip Strength and Incident All-Cause, Alzheimer, and Vascular Dementia
eTable 9. Linear Regression Coefficients for the Association of a 5-kg Decrement in Handgrip Strength With Normalized and Nonnormalized Total Brain, Hippocampal, and White Matter Hyperintensity Volume, Stratified by Gender
eTable 10. Age Less Than 65: Gender-Stratified Linear Regression Coefficients for a 5-kg Decrement in Handgrip Strength and Fluid Intelligence and Prospective Memory
eTable 11. Age Less Than 65: Linear Regression Coefficients for the Association of a 5-kg Decrement in Handgrip Strength with Normalized and Nonnormalized Total Brain, Hippocampal, and White Matter Hyperintensity Volume, Stratified by Gender
eTable 12. Age 65 and Older: Gender-Stratified Linear Regression Coefficients for a 5-kg Decrement in Handgrip Strength and Fluid Intelligence and Prospective Memory
eTable 13. Age 65 and Older: Gender-Stratified Hazard Ratios for a 5-kg Decrement in Handgrip Strength and Incident All-Cause, Alzheimer, and Vascular Dementia
eTable 14. Age 65 and Older: Linear Regression Coefficients for the Association of a 5-kg Decrement in Handgrip Strength With Normalized and Nonnormalized Total Brain, Hippocampal, and White Matter Hyperintensity Volume, Stratified by Gender
eTable 15. Gender-Stratified Hazard Ratios for the Association Between Handgrip Strength and Incident All-Cause Dementia Including Whether Job Involves Manual Labor as a Confounder in Models 2 and 3
eFigure 1. Example Kaplan-Meier Curves for All-Cause Dementia for the 5th Age-Decile in Men

© 2022 Duchowny KA et al. *JAMA Network Open.*

eFigure 2. Example Kaplan-Meier Curves for All-Cause Dementia for the 5th Age-Decile in Women
eFigure 3. Reverse Causation Schematic
eTable 16. Associations Between Polygenetic Risk Score (PGS), APOE*E4 Alleles and Handgrip Strength Stratified by Gender and Age

This supplemental material has been provided by the authors to give readers additional information about their work.

© 2022 Duchowny KA et al. *JAMA Network Open.*

eTable 1: Field IDs Used in Analyses

**52**	Month of birth
**31**	Sex
**189**	Townsend deprivation index at recruitment
**34**	Year of birth
**54**	UK Biobank assessment centre
**6138**	Qualifications
**21000**	Ethnic background
**3659**	Year immigrated to UK (United Kingdom)
**738**	Average total household income before tax
**21001**	Body mass index (BMI)
**21003**	Age when attended assessment centre
**53**	Date of attending assessment centre
**20018**	Prospective memory result
**20016**	Fluid intelligence score centre
**2178**	Overall health rating
**22009**	Genetic principal components
**884**	Number of days/week of moderate physical activity 10+ minutes
**130836**	Date F00 first reported (dementia in alzheimer's disease)
**130837**	Source of report of F00 (dementia in alzheimer's disease)
**130838**	Date F01 first reported (vascular dementia)
**130839**	Source of report of F01 (vascular dementia)
**130840**	Date F02 first reported (dementia in other diseases classified elsewhere)
**130841**	Source of report of F02 (dementia in other diseases classified elsewhere)
**130842**	Date F03 first reported (unspecified dementia)
**130843**	Source of report of F03 (unspecified dementia)
**131036**	Date G30 first reported (alzheimer's disease)
**131037**	Source of report of G30 (alzheimer's disease)
**42018**	Date of all cause dementia report
**42019**	Source of all cause dementia report
**42020**	Date of alzheimer's disease report
**42021**	Source of alzheimer's disease report
**42022**	Date of vascular dementia report
**42023**	Source of vascular dementia report
**42024**	Date of frontotemporal dementia report
**42025**	Source of frontotemporal dementia report
**25010**	Volume of brain, grey+white matter
**25009**	Volume of brain, grey+white matter (normalised for head size)

© 2022 Duchowny KA et al. *JAMA Network Open.*

**25019**	Volume of hippocampus (left)
**25020**	Volume of hippocampus (right)
**26521**	Volume of EstimatedTotalIntraCranial (whole brain)
**46**	Hand grip strength (left)
**47**	Hand grip strength (right)
**42040**	GP clinical event records
**42039**	GP prescription records
**42038**	GP registration records
**25781**	Total volume of white matter hyperintensities (from T1 and T2_FLAIR images)
**4079**	Diastolic blood pressure, automated reading
**94**	Diastolic blood pressure, manual reading
**4080**	Systolic blood pressure, automated reading
**93**	Systolic blood pressure, manual reading

© 2022 Duchowny KA et al. *JAMA Network Open.*

eTable 2: Read Codes for Dementia Diagnoses

'F110.' , 'XaIKB' , 'XaIKC' , 'Eu00.' , 'X002x' , 'X002x' , 'X002x' , 'X002x' , 'X002x' , 'X002x' , 'X002x' , 'X0030' , 'X0030' , 'X0030' , 'X0030' , 'X0030' , 'Eu00.' , 'X0030' , 'X0030' , 'Eu002' , 'Eu00z' , 'Eu00z' , 'Fyu30' , 'XE1Xs' , 'Xa0lH' , 'E0040' , 'E0041' , 'E0042' , 'E0043' , 'E004z' , 'XE1Xs' , 'XE1Xs' , 'XE1Xs' , 'X003R' , 'Xa0lH' , 'Xa0lH' , 'Xa0lH' , 'X003T' , 'X003V' , 'Eu01y' , 'Eu01z' , 'F21y2' , 'F21y2' , 'XaIRJ' , 'Eu020' , 'F111.' , 'A411.' , 'F11x7' , 'E011.' , 'E011.' , 'E011.' , 'E011.' , 'E0111' , 'E0112' , 'E011z' , 'XE1Xu' , 'X00Rk' , 'E0120' , 'Eu021' , 'XabVp' , 'XabVp' , 'Eu022' , 'Eu023' , 'X003P' , 'X003P' , 'XaOfZ' , 'Eu106' , 'Eu106' , 'Eu106' , 'E000.' , 'E001.' , 'E0010' , 'E0011' , 'E0012' , 'E0013' , 'E001z' , 'E002.' , 'E0020' , 'E0021' , 'E002z' , 'E003.' , 'XE1Xt' , 'X00R0' , 'E00z.' , 'E041.' , 'E04y.' , 'Eu02.' , 'XaKyY' , 'Eu02y' , 'X003A' , 'XE1Z6' , 'XE1Z6' , 'E00z.' , 'E00z.' , 'Eu02z' , 'Xa1GB' , 'Eu02z' , 'X00R2' , 'XE1Z6' , 'E00z.' , 'Eu02z' , 'XE1Xr' , 'Eu041' , 'F112.' , 'F10y.' , 'F10y0' , 'F10y1' , 'XaPws' , 'F10yz' , 'X0037' , 'F11..' , 'X003m' , 'F11yz' , 'Fyu31' , 'XE1Xr' , 'X00R2' , 'X002w' , '1461.' , 'XaaeA' , 'XaJBU' , 'XaJBV' , 'XaJBW' , 'XaJBX' , 'XaMJC' , 'XaMGF' , 'XabtQ' , 'Xaefu' , 'Xaefv' , 'XaaiW' , 'XacLx' , 'XabEl' , 'XaaBZ' , 'XacIx' , 'XacIy' , 'XacIz' , 'XacJ0' , 'XabEk' , 'XaYFR' , 'XabEi' , 'XacM2' , 'XaLFf' , 'XaLFo' , 'XaLFp' , 'XaMFy' , 'XaMG0' , 'XaMGG' , 'XaMGI' , 'XaMGJ' , 'XaMGK') OR read_2 IN ('F110.' , 'F1100' , 'F1101' , 'Eu00.' , 'Eu000' , 'Eu001' , 'Eu002' , 'Eu00z' , 'Fyu30' , 'E004.' , 'E0040' , 'E0041' , 'E0042' , 'E0043' , 'E004z' , 'Eu01.' , 'Eu010' , 'Eu011' , 'Eu012' , 'Eu013' , 'Eu01y' , 'Eu01z' , 'F21y2' , 'G678.' , 'Eu020' , 'F111.' , 'A411.' , 'F11x7' , 'E011.' , 'E0110' , 'E0111' , 'E0112' , 'E011z' , 'E012.' , 'E0120' , 'Eu021' , 'A4110' , 'Eu022' , 'Eu023' , 'Eu024' , 'F11x9' , 'Eu106' , 'E000.' , 'E001.' , 'E0010' , 'E0011' , 'E0012' , 'E0013' , 'E001z' , 'E002.' , 'E0020' , 'E0021' , 'E002z' , 'E003.' , 'E00y.' , 'E00z.' , 'E041.' , 'E04y.' , 'Eu02.' , 'Eu025' , 'Eu02y' , 'F116.' , 'Eu02z' , 'Eu041' , 'F112.' , 'F10y.' , 'F10y0' , 'F10y1' , 'F10y2' , 'F10yz' , 'F118.' , 'F11y.' , 'F11y2' , 'F11yz' , 'Fyu31' , 'E00..' , '1461.' , '38C13' , '3AE3.' , '3AE4.' , '3AE5.' , '3AE6.' , '66h..' , '6AB..' , '8BM02' , '8BM50' , '8BM60' , '8BPa.' , '8CMe0' , '8CMG2' , '8CMZ.' , '8CMZ0' , '8CMZ1' , '8CMZ2' , '8CMZ3' , '8CSA.' , '8Hla.' , '8IAe0' , '8IAe2' , '9hD..' , '9hD0.' , '9hD1.' , '9Ou..' , '9Ou1.' , '9Ou2.' , '9Ou3.' , '9Ou4.' , '9Ou5.'

© 2022 Duchowny KA et al. *JAMA Network Open.*

eMethods: Primary Outcomes

Cognitive Outcomes. Fluid intelligence, a 13-item task of problem-solving requiring logic and reasoning ability, was evaluated using a touch screen at UKB assessment center visits.[13] This measure was available for all participants. Prospective Memory, a type of episodic memory, measures an individual's memory for future tasks and was assessed via touchscreen. Prior to other cognitive assessments, participants were given an instruction that they were later asked to recall after other cognitive assessments were complete (i.e., "At the end of the games we will show you four coloured shapes and ask you to touch the Blue Square. However, to test your memory, we want you to actually touch the Orange Circle instead"). Three possible scores are obtained: 0- Instruction not recalled, either skipped or incorrect; 1 - correct recall on first attempt; 2 - correct recall on second attempt. Values were coded as 1 for correct on the first attempt, and 0 otherwise.

Incident Dementia. Dementia diagnosis was ascertained using a combination of diagnoses obtained from primary care, hospital inpatient, death registered records, and self-report. Algorithmically defined dementia and First Occurrences Dementia and primary care records were merged. Only individuals with a confirmed primary care linkage were included in analyses with this outcome. We used the date of diagnosis at the earliest date of dementia codes recorded, irrespective of the source used. Similar merging with primary care records was performed for Alzheimer's disease and vascular dementia Primary care records we queried with the following ICD10 and ICD9 codes for dementia, Alzheimer's disease, and vascular dementia . Dementia: F02, F03, G31, 331.0, 294.1, and 294.8, plus codes for Alzheimer's disease and vascular dementia. Alzheimer's disease: F00, G30, and 331.0. Vascular dementia: F01, and 290.4. Participant time in years was calculated from the date of handgrip strength assessment until the date of dementia diagnosis, date of loss to follow-up, date of death, censoring (December 4, 2020), or updating date of HGS.

Neuroimaging Outcomes. We utilized global and regional brain Imaging Derived Phenotypes (IDPs) provided by the UK Biobank brain imaging team. Details on imaging acquisition and the imaging processing are available elsewhere.[13] The neuroimaging outcomes of interest for this study included: Total brain volume (TBV), defined as the sum of white and grey matter independent of cerebrospinal fluid, was ascertained using T1 structural brain MRI. (Analyses of TBV normalized for head size (Field 25009) are included in the appendix.) Hippocampal Volume was assessed using the T1 structural brain MRI. Main analyses consider total hippocampal volume while sensitivity analyses consider the right and left hippocampus separately. White matter hyperintensity volume (WMH) was ascertained using T1-weighted and T2 FLAIR volumes structural brain MRI (field 25781). Main analyses used total brain volume and hippocampal volume scaled by intracranial volume, such that scaled volume is given by crude volume divided by intracranial volume times mean intracranial volume. Appendix 3 provides results of these analyses using crude volumes. Given known differences in head size, we report all gender-stratified model results normalized for intracranial volume in Figure 4 and non-normalized results in the Appendix 4.

© 2022 Duchowny KA et al. *JAMA Network Open.*

eMethods: Covariate Categorizations

Collapsed Race/Ethnicity Categories
White: British, White, Irish, Any other white background
Black: Black or Black British, White and Black Caribbean, White and Black African, African, Caribbean, Any other Black background
Asian: Asian or Asian British, Chinese, Indian, Pakistani, Bangladeshi, Any other Asian background
Mixed/Other: Mixed, Any other mixed background, Other ethnic group

High School or Equivalent Educational Categories
College or University degree
A levels/AS levels or equivalent
NVQ or HND or HNC or equivalent
Other professional qualifications e.g.: nursing, teaching
A levels/AS levels or equivalent

© 2022 Duchowny KA et al. *JAMA Network Open.*

eMethods: Statistical Modeling Approach

Cox proportional hazard models were used to determine the relative hazard of dementia associated with decreased HGS, accounting for repeated HGS measures. Death, as obtained from the death registry data, was considered a competing event. Cox proportional hazard assumptions were met for all-cause dementia models at a significance threshold of 0.05 (see Appendix X).[19] Stratification variables were selected in order to meet the proportionality assumption, which included stratification by age decile (all models), number of APOE-e4 alleles, and race (confounder and full models), and quartile of systolic blood pressure (full model). Overall health rating (good and excellent vs. other categories) and assessment centre (London area vs. other areas) were dichotomized due to no dementia diagnosis events within some strata of those variables. We employed generalized linear or logistic mixed effects models to examine the association between HGS and cognitive outcomes and MRI volumetric measures. All mixed effects models included a random intercept and fixed slope and adjusted for age and age squared as orthogonal polynomials.

© 2022 Duchowny KA et al. *JAMA Network Open.*

eTable 3: Missingness in First Visit for the Entire Sample

	**Total (N=497965)**
No GP Registration	271152 (54.5%)
Missing HGS at BL	0 (0%)
Missing Assessment Center	0 (0%)
Missing TDI	614 (0.1%)
Missing Race	2365 (0.5%)
Missing Education	4238 (0.9%)
Missing APOE-e4	14016 (2.8%)
Missing Age	0 (0%)
Missing BMI	1183 (0.2%)
Missing Physical Activity	26465 (5.3%)
Missing Overall Health Rating	542 (0.1%)
Missing SBP	33364 (6.7%)
Missing Gender	0 (0%)

© 2022 Duchowny KA et al. *JAMA Network Open.*

Additional Summaries of Sample Characteristics

eTable 4: Demographic Characteristics Among Individuals Completing Cognitive Testing

	Women (N=82363)	Men (N=71034)	Total (N=153397)
	**Mean (SD) or N(%)**	**Mean (SD) or N(%)**	**Mean (SD) or N(%)**
**Age**	56.43 (8.1)	57.00 (8.3)	56.69 (8.2)
**High School Equivalent**			
Yes	54373 (66.0%)	50142 (70.6%)	104515 (68.1%)
No	27990 (34.0%)	20892 (29.4%)	48882 (31.9%)
**Race**			
Asian	2745 (3.3%)	2876 (4.0%)	5621 (3.7%)
Black	2478 (3.0%)	1705 (2.4%)	4183 (2.7%)
Mixed	1322 (1.6%)	862 (1.2%)	2184 (1.4%)
White	75818 (92.1%)	65591 (92.3%)	141409 (92.2%)
**BMI**	26.94 (5.1)	27.78 (4.2)	27.33 (4.7)
**Townsend Deprivation Index**	-1.20 (2.9)	-1.17 (3.0)	-1.18 (2.9)
**Days per week of moderate physical activity**	3.79 (2.3)	3.69 (2.3)	3.75 (2.3)
**Systolic blood pressure**	138.03 (20.4)	142.98 (18.5)	140.32 (19.7)
**Overall health rating**			
Prefer not to answer	25 (0.0%)	25 (0.0%)	50 (0.0%)
Do not know	238 (0.3%)	190 (0.3%)	428 (0.3%)
Excellent	12473 (15.1%)	9769 (13.8%)	22242 (14.5%)
Good	49826 (60.5%)	40146 (56.5%)	89972 (58.7%)
Fair	16559 (20.1%)	17297 (24.4%)	33856 (22.1%)
Poor	3242 (3.9%)	3607 (5.1%)	6849 (4.5%)
**APOE-e4 Carrier**			
Yes	23138 (28.1%)	19911 (28.0%)	43049 (28.1%)
No	59225 (71.9%)	51123 (72.0%)	110348 (71.9%)
**Baseline HGS**	24.49 (6.3)	40.63 (8.9)	31.97 (11.1)

© 2022 Duchowny KA et al. *JAMA Network Open.*

eTable 5: Demographic Characteristics Among Individuals With Neuroimaging Measures

	**Women (N=20204)**	**Men (N=18439)**	**Total (N=38643)**
	**Mean (SD) or N(%)**	**Mean (SD) or N(%)**	**Mean (SD) or N(%)**
**Age**	54.31 (7.4)	55.66 (7.6)	54.95 (7.5)
**High School Equivalent**			
Yes	15794 (78.2%)	15298 (83.0%)	31092 (80.5%)
No	4410 (21.8%)	3141 (17.0%)	7551 (19.5%)
**Race**			
Asian	238 (1.2%)	315 (1.7%)	553 (1.4%)
Black	183 (0.9%)	126 (0.7%)	309 (0.8%)
Mixed	150 (0.7%)	101 (0.5%)	251 (0.6%)
White	19633 (97.2%)	17897 (97.1%)	37530 (97.1%)
**BMI**	25.98 (4.5)	27.07 (3.7)	26.50 (4.2)
**Townsend Deprivation Index**	-1.87 (2.7)	-1.96 (2.7)	-1.91 (2.7)
**Days per week of moderate physical activity**	3.55 (2.3)	3.42 (2.2)	3.49 (2.3)
**Systolic blood pressure**	133.14 (19.1)	140.50 (17.4)	136.65 (18.7)
**Overall health rating**			
Prefer not to answer	4 (0.0%)	1 (0.0%)	5 (0.0%)
Do not know	26 (0.1%)	20 (0.1%)	46 (0.1%)
Excellent	5021 (24.9%)	4248 (23.0%)	9269 (24.0%)
Good	12411 (61.4%)	11079 (60.1%)	23490 (60.8%)
Fair	2406 (11.9%)	2777 (15.1%)	5183 (13.4%)
Poor	336 (1.7%)	314 (1.7%)	650 (1.7%)
**APOE-e4 Carrier**			
Yes	5680 (28.1%)	4971 (27.0%)	10651 (27.6%)
No	14524 (71.9%)	13468 (73.0%)	27992 (72.4%)
**Baseline HGS**	26.63 (6.1)	43.47 (8.5)	34.67 (11.2)

eTable 6: Demographic Characteristics Among Individuals With Gp Linkages, Stratified by Median HGS.

	**Women**		**Men**		**Total**	
	**At or Above Median (N=51428)**	**Below Median (N=51307)**	**At or Above Median (N=45338)**	**Below Median (N=42333)**	**At or Above Median (N=96766)**	**Below Median (N=93640)**
**Age**	54.01 (7.9)	58.53 (7.3)	54.87 (8.1)	58.67 (7.8)	54.42 (8.0)	58.59 (7.5)
**High School Equivalent**						
Yes	35740 (69.5%)	29369 (57.2%)	33017 (72.8%)	27539 (65.1%)	68757 (71.1%)	56908 (60.8%)
No	15688 (30.5%)	21938 (42.8%)	12321 (27.2%)	14794 (34.9%)	28009 (28.9%)	36732 (39.2%)
**Race**						
Asian	580 (1.1%)	1464 (2.9%)	598 (1.3%)	1626 (3.8%)	1178 (1.2%)	3090 (3.3%)
Black	847 (1.6%)	541 (1.1%)	591 (1.3%)	410 (1.0%)	1438 (1.5%)	951 (1.0%)
Mixed	424 (0.8%)	538 (1.0%)	285 (0.6%)	429 (1.0%)	709 (0.7%)	967 (1.0%)
White	49577 (96.4%)	48764 (95.0%)	43864 (96.7%)	39868 (94.2%)	93441 (96.6%)	88632 (94.7%)
**BMI**	26.86 (5.0)	27.24 (5.2)	27.95 (4.0)	27.77 (4.4)	27.37 (4.6)	27.48 (4.8)
**Townsend Deprivation Index**	-1.62 (2.8)	-1.30 (3.0)	-1.68 (2.9)	-1.12 (3.1)	-1.65 (2.8)	-1.22 (3.0)
**Days per week of moderate physical activity**	3.65 (2.3)	3.62 (2.4)	3.67 (2.3)	3.58 (2.4)	3.66 (2.3)	3.60 (2.4)
**Systolic blood pressure**	136.03 (19.9)	138.59 (20.4)	143.01 (18.0)	143.04 (19.0)	139.30 (19.3)	140.60 (19.9)
**Overall health rating**						
Prefer not to answer	9 (0.0%)	18 (0.0%)	7 (0.0%)	19 (0.0%)	16 (0.0%)	37 (0.0%)
Do not know	128 (0.2%)	199 (0.4%)	116 (0.3%)	163 (0.4%)	244 (0.3%)	362 (0.4%)
Excellent	10454 (20.3%)	6926 (13.5%)	8057 (17.8%)	5478 (12.9%)	18511 (19.1%)	12404 (13.2%)
Good	31682 (61.6%)	29998 (58.5%)	26426 (58.3%)	23140 (54.7%)	58108 (60.1%)	53138 (56.7%)

© 2022 Duchowny KA et al. *JAMA Network Open.*

Fair	8009 (15.6%)	11492 (22.4%)	9232 (20.4%)	10698 (25.3%)	17241 (17.8%)	22190 (23.7%)
Poor	1146 (2.2%)	2674 (5.2%)	1500 (3.3%)	2835 (6.7%)	2646 (2.7%)	5509 (5.9%)
**APOE-e4 Carrier**						
Yes	14754 (28.7%)	14348 (28.0%)	12960 (28.6%)	11916 (28.1%)	27714 (28.6%)	26264 (28.0%)
No	36674 (71.3%)	36959 (72.0%)	32378 (71.4%)	30417 (71.9%)	69052 (71.4%)	67376 (72.0%)
**Dementia Dx in Follow-Up**						
Yes	358 (0.7%)	855 (1.7%)	427 (0.9%)	996 (2.4%)	785 (0.8%)	1851 (2.0%)
No	51070 (99.3%)	50452 (98.3%)	44911 (99.1%)	41337 (97.6%)	95981 (99.2%)	91789 (98.0%)

© 2022 Duchowny KA et al. *JAMA Network Open.*

Regression Coefficients for a 5-kg Decrement in Hand-Grip Strength

Note: All tables give results for models 1, 2, and 3 in the corresponding columns.

eTable 7: Gender-stratified linear regression coefficients for a 5-kg decrement in hand-grip strength and fluid intelligence and prospective memory.

	Model 1	Model 2	Model 3
Men: Fluid Intelligence	-0.028 (-0.032, -0.024)	-0.006 (-0.010, -0.002)	-0.007 (-0.010, -0.003)
	p < 0.001	p = 0.002	p = 0.0003
Observations	95,935	95,935	95,935
Women: Fluid Intelligence	-0.068 (-0.073, -0.064)	-0.047 (-0.051, -0.042)	-0.043 (-0.047, -0.038)
	p < 0.001	p < 0.001	p < 0.001
Observations	108,002	108,002	108,002
Men: Prospective Memory	0.876 (0.867, 0.886)	0.912 (0.902, 0.922)	0.910 (0.900, 0.921)
	p < 0.001	p < 0.001	p < 0.001
Observations	98,502	98,502	98,502
Women: Prospective Memory	0.851 (0.839, 0.864)	0.880 (0.867, 0.894)	0.888 (0.874, 0.901)
	p < 0.001	p < 0.001	p < 0.001
Observations	110,609	110,609	110,609

eTable 8: Gender-Stratified Hazard Ratios for the Association Between Handgrip Strength and Incident All-Cause, Alzheimer, and Vascular Dementia.

	Model 1	Model 2	Model 3
Men: All-Cause Dementia	1.18 (1.14, 1.21)	1.16 (1.12, 1.20)	1.14 (1.10, 1.18)
	p < 0.001	p < 0.001	p < 0.0010.00
Observations	100,384	100,384	100,384
Women: All-Cause Dementia	1.17 (1.12, 1.23)	1.14 (1.09, 1.20)	1.10 (1.05, 1.15)
	p = 0. p < 0.001	p < 0.001	p < 0.001
Observations	115,787	115,787	115,787
Men: Alzheimer's Disease	1.11 (1.06, 1.17)	1.11 (1.05, 1.16)	1.10 (1.04, 1.15)
	p < 0.001	p < 0.001	p < 0.001
Observations	100,384	100,384	100,384
Women: Alzheimer's Disease	1.15 (1.07, 1.24)	1.13 (1.05, 1.21)	1.10 (1.02, 1.18)
	p < 0.001	p = 0.002	p = 0.02
Observations	115,787	115,787	115,787
Men: Vascular Dementia	1.24 (1.17, 1.32)	1.23 (1.15, 1.31)	1.20 (1.12, 1.28)
	p < 0.001	p < 0.001	p < 0.001
Observations	100,384	100,384	100,384
Women: Vascular Dementia	1.24 (1.11, 1.39)	1.20 (1.07, 1.35)	1.12 (1.00, 1.26)
	p < 0.001	p = 0.002	p = 0.06
Observations	115,787	115,787	115,787

© 2022 Duchowny KA et al. *JAMA Network Open.*

eTable 9: Linear Regression Coefficients for the Association of a 5-kg Decrement in Handgrip Strength With Normalized and Nonnormalized Total Brain, Hippocampal, and White Matter Hyperintensity Volume, Stratified by Gender. *TBV: total brain volume; HV: hippocampal volume; HVR: hippocampal volume, right; HVL: hippocampal volume, left; WMHI: white matter hyperintensity volume.*

	Model 1	Model 2	Model 3
Men: Crude TBV	-5,879.71 (-6,731.46, -5,027.95)	-5,163.70 (-6,012.91, -4,314.48)	-5,110.34 (-5,966.62, -4,254.07)
	p < 0.001	p < 0.001	p < 0.001
Observations	18,438	18,438	18,438
Women: Crude TBV	-7,064.74 (-8,088.62, -6,040.87)	-6,760.95 (-7,794.56, -5,727.34)	-6,747.66 (-7,791.96, -5,703.35)
	p < 0.001	p < 0.001	p < 0.001
Observations	20,204	20,204	20,204
Men: Normalized TBV	-255.20 (-660.49, 150.09)	-223.67 (-630.73, 183.38)	-322.42 (-732.56, 87.72)
	p = 0.22	p = 0.29	p = 0.13
Observations	18,438	18,438	18,438
Women: Normalized TBV	630.19 (111.71, 1,148.67)	329.23 (-196.28, 854.73)	296.09 (-235.73, 827.91)
	p = 0.02	p = 0.22	p = 0.28
Observations	20,204	20,204	20,204
Men: Normalized HV	3.97 (-3.90, 11.84)	0.27 (-7.68, 8.23)	-0.64 (-8.67, 7.39)
	p = 0.33	p = 0.95	p = 0.88
Observations	18,438	18,438	18,438
Women: Normalized HV	13.74 (3.90, 23.57)	9.35 (-0.70, 19.40)	10.27 (0.10, 20.43)
	p = 0.01	p = 0.07	p = 0.05
Observations	20,204	20,204	20,204

© 2022 Duchowny KA et al. *JAMA Network Open.*

	Model 1	Model 2	Model 3
Men: Crude HV	-33.20 (-41.12, -25.29)	-32.44 (-40.44, -24.44)	-32.34 (-40.42, -24.26)
	p < 0.001	p < 0.001	p < 0.001
Observations	18,438	18,438	18,438
Women: Crude HV	-39.12 (-48.32, -29.92)	-39.33 (-48.70, -29.95)	-38.27 (-47.76, -28.79)
	p < 0.001	p < 0.001	p < 0.001
Observations	20,204	20,204	20,204
Men: Crude HVL	-17.37 (-21.75, -13.00)	-16.94 (-21.37, -12.51)	-17.13 (-21.60, -12.66)
	p < 0.001	p < 0.001	p < 0.001
Observations	18,438	18,438	18,438
Women: Crude HVL	-17.12 (-22.23, -12.01)	-17.11 (-22.31, -11.90)	-16.80 (-22.07, -11.53)
	p < 0.001	p < 0.001	p < 0.001
Observations	20,204	20,204	20,204
Men: Crude HVR	-15.83 (-20.37, -11.30)	-15.51 (-20.10, -10.92)	-15.22 (-19.86, -10.58)
	p < 0.001	p < 0.001	p < 0.001
Observations	18,438	18,438	18,438
Women: Crude HVR	-21.98 (-27.14, -16.82)	-22.21 (-27.47, -16.95)	-21.46 (-26.79, -16.14)
	p < 0.001	p < 0.001	p < 0.001
Observations	20,204	20,204	20,204
Men: Normalized HVL	1.12 (-3.21, 5.46)	-0.63 (-5.02, 3.75)	-1.30 (-5.72, 3.13)
	p = 0.62	p = 0.78	p = 0.57
Observations	18,438	18,438	18,438
Women: Normalized HVL	8.96 (3.46, 14.46)	6.90 (1.27, 12.52)	7.18 (1.49, 12.88)
	p = 0.002	p = 0.02	p = 0.02
Observations	20,204	20,204	20,204

© 2022 Duchowny KA et al. *JAMA Network Open.*

	Model 1	Model 2	Model 3
Men: Normalized HVR	2.85 (-1.56, 7.25)	0.91 (-3.55, 5.37)	0.66 (-3.84, 5.17)
	p = 0.21	p = 0.70	p = 0.78
Observations	18,438	18,438	18,438
Women: Normalized HVR	4.77 (-0.65, 10.19)	2.45 (-3.10, 7.99)	3.07 (-2.54, 8.69)
	p = 0.09	p = 0.39	p = 0.29
Observations	20,204	20,204	20,204
Men: WMHI	23.19 (-37.12, 83.50)	49.42 (-11.67, 110.51)	92.22 (31.09, 153.35)
	p = 0.46	p = 0.12	p = 0.004
Observations	18,438	18,438	18,438
Women: WMHI	41.32 (-26.99, 109.63)	72.46 (2.78, 142.14)	83.56 (13.54, 153.58)
	p = 0.24	p = 0.05	p = 0.02
Observations	20,204	20,204	20,204

© 2022 Duchowny KA et al. *JAMA Network Open.*

eTable 10: Age Less Than 65: Gender-Stratified Linear Regression Coefficients for a 5-kg Decrement in Hand-Grip Strength and Fluid Intelligence and Prospective Memory.

	Model 1	Model 2	Model 3
Men: Fluid Intelligence	-0.025 (-0.029, -0.021)	-0.002 (-0.007, 0.002)	-0.004 (-0.008, 0.001)
	p < 0.001	p = 0.266	p = 0.085
Observations	67,472	67,472	67,472
Women: Fluid Intelligence	-0.073 (-0.078, -0.067)	-0.050 (-0.055, -0.045)	-0.046 (-0.051, -0.041)
	p < 0.001	p < 0.001	p < 0.001
Observations	82,031	82,031	82,031
Men: Prospective Memory	0.870 (0.858, 0.881)	0.913 (0.901, 0.926)	0.911 (0.899, 0.924)
	p < 0.001	p < 0.001	p < 0.001
Observations	69,028	69,028	69,028
Women: Prospective Memory	0.843 (0.829, 0.858)	0.878 (0.862, 0.893)	0.883 (0.868, 0.899)
	p < 0.001	p < 0.001	p < 0.001
Observations	83,701	83,701	83,701

© 2022 Duchowny KA et al. *JAMA Network Open.*

eTable 11: Age Less Than 65: Linear Regression Coefficients for the Association of a 5-kg Decrement in Handgrip Strength With Normalized and Nonnormalized Total Brain, Hippocampal, and White Matter Hyperintensity Volume, Stratified by Gender. *TBV: total brain volume; HV: hippocampal volume; HVR: hippocampal volume, right; HVL: hippocampal volume, left; WMHI: white matter hyperintensity volume.*

	Model 1	Model 2	Model 3
Men: Crude TBV	-5,951.13 (-6,857.86, -5,044.40)	-5,219.49 (-6,123.92, -4,315.07)	-5,138.99 (-6,051.61, -4,226.38)
	p < 0.001	p < 0.001	p < 0.001
Observations	16,088	16,088	16,088
Women: Crude TBV	-6,991.77 (-8,063.81, -5,919.72)	-6,694.68 (-7,776.43, -5,612.92)	-6,669.55 (-7,763.02, -5,576.08)
	p < 0.001	p < 0.001	p < 0.001
Observations	18,475	18,475	18,475
Men: Normalized TBV	-237.75 (-671.22, 195.72)	-205.72 (-641.81, 230.37)	-331.53 (-771.12, 108.06)
	p = 0.29	p = 0.36	p = 0.14
Observations	16,088	16,088	16,088
Women: Normalized TBV	805.56 (267.24, 1,343.87)	482.38 (-63.86, 1,028.63)	426.62 (-126.26, 979.50)
	p = 0.004	p = 0.09	p = 0.14
Observations	18,475	18,475	18,475

© 2022 Duchowny KA et al. *JAMA Network Open.*

	Model 1	Model 2	Model 3
Men: Normalized HV	3.84 (-4.44, 12.11)	0.26 (-8.12, 8.64)	-0.77 (-9.23, 7.70)
	p = 0.37	p = 0.96	p = 0.86
Observations	16,088	16,088	16,088
Women: Normalized HV	16.92 (6.78, 27.05)	12.31 (1.94, 22.67)	13.35 (2.86, 23.84)
	p = 0.002	p = 0.02	p = 0.02
Observations	18,475	18,475	18,475
Men: Crude HV	-34.08 (-42.43, -25.73)	-33.10 (-41.55, -24.65)	-32.73 (-41.27, -24.19)
	p < 0.001	p < 0.001	p < 0.001
Observations	16,088	16,088	16,088
Women: Crude HV	-37.00 (-46.56, -27.44)	-37.31 (-47.06, -27.56)	-35.93 (-45.80, -26.06)
	p < 0.001	p < 0.001	p < 0.001
Observations	18,475	18,475	18,475
Men: Crude HVL	-17.76 (-22.39, -13.13)	-17.17 (-21.86, -12.49)	-17.18 (-21.92, -12.45)
	p < 0.001	p < 0.001	p < 0.001
Observations	16,088	16,088	16,088
Women: Crude HVL	-16.04 (-21.36, -10.72)	-16.05 (-21.48, -10.62)	-15.64 (-21.14, -10.14)
	p < 0.001	p < 0.001	p < 0.001
Observations	18,475	18,475	18,475
Men: Crude HVR	-16.33 (-21.12, -11.54)	-15.93 (-20.78, -11.08)	-15.55 (-20.46, -10.65)
	p < 0.001	p < 0.001	p < 0.001
Observations	16,088	16,088	16,088
Women: Crude HVR	-20.94 (-26.30, -15.58)	-21.25 (-26.72, -15.78)	-20.29 (-25.83, -14.74)
	p < 0.001	p < 0.001	p < 0.001
Observations	18,475	18,475	18,475

© 2022 Duchowny KA et al. *JAMA Network Open.*

	Model 1	Model 2	Model 3
Men: Normalized HVL	1.09 (-3.48, 5.66)	-0.57 (-5.20, 4.06)	-1.25 (-5.93, 3.43)
	p = 0.65	p = 0.81	p = 0.60
Observations	16,088	16,088	16,088
Women: Normalized HVL	10.61 (4.91, 16.31)	8.46 (2.63, 14.30)	8.76 (2.85, 14.66)
	p < 0.001	p = 0.005	p = 0.004
Observations	18,475	18,475	18,475
Men: Normalized HVR	2.75 (-1.89, 7.39)	0.84 (-3.87, 5.54)	0.49 (-4.26, 5.24)
	p = 0.25	p = 0.73	p = 0.84
Observations	16,088	16,088	16,088
Women: Normalized HVR	6.30 (0.71, 11.89)	3.83 (-1.89, 9.56)	4.58 (-1.21, 10.38)
	p = 0.03	p = 0.19	p = 0.13
Observations	18,475	18,475	18,475
Men: WMHI	18.30 (-37.03, 73.62)	38.87 (-17.22, 94.97)	81.29 (25.21, 137.37)
	p = 0.52	p = 0.18	p = 0.005
Observations	16,088	16,088	16,088
Women: WMHI	66.28 (2.15, 130.42)	90.85 (25.40, 156.30)	102.21 (36.48, 167.95)
	p = 0.05	p = 0.01	p = 0.003
Observations	18,475	18,475	18,475

© 2022 Duchowny KA et al. *JAMA Network Open.*

eTable 12: Age 65 and Older: Gender-Stratified Linear Regression Coefficients for a 5-kg Decrement in Hand-Grip Strength and Fluid Intelligence and Prospective Memory.

	Model 1	Model 2	Model 3
Men: Fluid Intelligence	-0.037 (-0.044, -0.030)	-0.014 (-0.020, -0.007)	-0.013 (-0.020, -0.007)
	p < 0.001	p < 0.001	p = 0.0002
Observations	28,463	28,463	28,463
Women: Fluid Intelligence	-0.063 (-0.073, -0.054)	-0.040 (-0.049, -0.031)	-0.037 (-0.046, -0.027)
	p < 0.001	p < 0.001	p < 0.001
Observations	25,971	25,971	25,971
Men: Prospective Memory	0.892 (0.875, 0.909)	0.910 (0.892, 0.929)	0.911 (0.892, 0.929)
	p < 0.001	p < 0.001	p < 0.001
Observations	29,474	29,474	29,474
Women: Prospective Memory	0.875 (0.851, 0.899)	0.890 (0.864, 0.916)	0.900 (0.874, 0.926)
	p < 0.001	p < 0.001	p < 0.001
Observations	26,908	26,908	26,908

© 2022 Duchowny KA et al. *JAMA Network Open.*

eTable 13: Age 65 and Older: Gender-Stratified Hazard Ratios for a 5-kg Decrement in Hand-Grip Strength and Incident All-Cause, Alzheimer, and Vascular Dementia.

	Model 1	Model 2	Model 3
Men: All-Cause Dementia	1.16 (1.12, 1.21)	1.15 (1.10, 1.20)	1.14 (1.09, 1.19)
	$p < 0.001$	$p < 0.001$	$p < 0.001$
Observations	24,287	24,287	24,287
Women: All-Cause Dementia	1.14 (1.06, 1.21)	1.12 (1.05, 1.20)	1.08 (1.01, 1.15)
	$p = 0.0002$	$p = 0.001$	$p = 0.04$
Observations	23,242	23,242	23,242
Men: Alzheimer's Disease	1.10 (1.03, 1.18)	1.10 (1.03, 1.18)	1.10 (1.02, 1.18)
	$p = 0.004$	$p = 0.01$	$p = 0.02$
Observations	24,287	24,287	24,287
Women: Alzheimer's Disease	1.15 (1.04, 1.26)	1.13 (1.03, 1.25)	1.10 (1.00, 1.22)
	$p = 0.005$	$p = 0.02$	$p = 0.05$
Observations	23,242	23,242	23,242
Men: Vascular Dementia	1.18 (1.09, 1.28)	1.18 (1.09, 1.28)	1.17 (1.07, 1.28)
	$p = 0.0001$	$p = 0.0002$	$p = 0.0004$
Observations	24,287	24,287	24,287
Women: Vascular Dementia	1.17 (1.02, 1.35)	1.14 (0.99, 1.32)	1.08 (0.94, 1.25)
	$p = 0.03$	$p = 0.07$	$p = 0.29$
Observations	23,242	23,242	23,242

© 2022 Duchowny KA et al. *JAMA Network Open.*

eTable 14: Age 65 and Older: Linear Regression Coefficients for the Association of a 5-kg Decrement in Handgrip Strength With Normalized and Nonnormalized Total Brain, Hippocampal, and White Matter Hyperintensity Volume, Stratified by Gender. *TBV: total brain volume; HV: hippocampal volume; HVR: hippocampal volume, right; HVL: hippocampal volume, left; WMHI: white matter hyperintensity volume.*

	Model 1	Model 2	Model 3
Men: Crude TBV	-5,249.93 (-7,749.19, -2,750.67)	-4,533.32 (-7,033.07, -2,033.56)	-4,684.67 (-7,201.14, -2,168.21)
	p = 0.0001	p = 0.0004	p = 0.0003
Observations	2,350	2,350	2,350
Women: Crude TBV	-7,997.20 (-11,446.90, -4,547.49)	-7,359.67 (-10,909.58, -3,809.76)	-7,385.65 (-10,954.15, -3,817.16)
	p = 0.0000	p = 0.0001	p = 0.0001
Observations	1,729	1,729	1,729
Men: Normalized TBV	-388.77 (-1,533.93, 756.40)	-237.01 (-1,378.18, 904.16)	-121.27 (-1,270.09, 1,027.55)
	p = 0.51	p = 0.69	p = 0.84
Observations	2,350	2,350	2,350
Women: Normalized TBV	-1,482.83 (-3,383.00, 417.34)	-1,540.45 (-3,477.77, 396.88)	-1,258.34 (-3,216.14, 699.46)
	p = 0.13	p = 0.12	p = 0.21
Observations	1,729	1,729	1,729

© 2022 Duchowny KA et al. *JAMA Network Open.*

	Model 1	Model 2	Model 3
Men: Normalized HV	5.52 (-19.50, 30.53)	1.94 (-23.24, 27.11)	2.60 (-22.76, 27.95)
	p = 0.67	p = 0.89	p = 0.85
Observations	2,350	2,350	2,350
Women: Normalized HV	-23.30 (-61.95, 15.35)	-19.76 (-59.44, 19.92)	-20.87 (-60.97, 19.23)
	p = 0.24	p = 0.33	p = 0.31
Observations	1,729	1,729	1,729
Men: Crude HV	-25.32 (-49.90, -0.75)	-25.21 (-50.12, -0.30)	-26.23 (-51.36, -1.10)
	p = 0.05	p = 0.05	p = 0.05
Observations	2,350	2,350	2,350
Women: Crude HV	-64.01 (-97.69, -30.34)	-56.15 (-90.68, -21.62)	-59.02 (-93.94, -24.11)
	p = 0.0002	p = 0.002	p = 0.001
Observations	1,729	1,729	1,729
Men: Crude HVL	-13.90 (-27.22, -0.57)	-13.86 (-27.39, -0.34)	-14.74 (-28.39, -1.09)
	p = 0.05	p = 0.05	p = 0.04
Observations	2,350	2,350	2,350
Women: Crude HVL	-29.56 (-47.73, -11.39)	-25.57 (-44.19, -6.94)	-26.81 (-45.65, -7.98)
	p = 0.002	p = 0.01	p = 0.01
Observations	1,729	1,729	1,729
Men: Crude HVR	-11.43 (-25.46, 2.61)	-11.35 (-25.59, 2.90)	-11.49 (-25.87, 2.88)
	p = 0.12	p = 0.12	p = 0.12
Observations	2,350	2,350	2,350
Women: Crude HVR	-34.43 (-53.18, -15.67)	-30.56 (-49.91, -11.22)	-32.19 (-51.75, -12.64)
	p = 0.0004	p = 0.002	p = 0.002
Observations	1,729	1,729	1,729

© 2022 Duchowny KA et al. *JAMA Network Open.*

	Model 1	Model 2	Model 3
Men: Normalized HVL	1.61 (-11.89, 15.10)	-0.13 (-13.75, 13.50)	-0.12 (-13.84, 13.61)
	p = 0.82	p = 0.99	p = 0.99
Observations	2,350	2,350	2,350
Women: Normalized HVL	-10.03 (-30.74, 10.68)	-7.98 (-29.23, 13.28)	-8.28 (-29.76, 13.19)
	p = 0.35	p = 0.47	p = 0.45
Observations	1,729	1,729	1,729
Men: Normalized HVR	3.89 (-10.04, 17.82)	2.05 (-12.00, 16.10)	2.71 (-11.44, 16.85)
	p = 0.59	p = 0.78	p = 0.71
Observations	2,350	2,350	2,350
Women: Normalized HVR	-13.28 (-34.48, 7.91)	-11.79 (-33.65, 10.08)	-12.60 (-34.69, 9.50)
	p = 0.22	p = 0.30	p = 0.27
Observations	1,729	1,729	1,729
Men: WMHI	55.97 (-245.23, 357.17)	130.11 (-174.84, 435.07)	178.70 (-126.32, 483.72)
	p = 0.72	p = 0.41	p = 0.26
Observations	2,350	2,350	2,350
Women: WMHI	-238.23 (-653.51, 177.04)	-121.82 (-553.63, 309.99)	-116.93 (-550.69, 316.82)
	p = 0.27	p = 0.59	p = 0.60
Observations	1,729	1,729	1,729

© 2022 Duchowny KA et al. *JAMA Network Open.*

eTable 15: Gender-Stratified Hazard Ratios for the Association Between Handgrip Strength and Incident All-Cause Dementia Including Whether Job Involves Manual Labor as a Confounder in Models 2 and 3. Extent of manual labor was classified as always, usually, sometimes, and rarely/never (field 816). Model 1 with the same number of observations as complete case counts used for models 2 and 3 is given for comparison.

	Model 1	Model 2	Model 3
Men: All-Cause Dementia	1.14 (1.07, 1.21)	1.12 (1.05, 1.19)	1.11 (1.04, 1.18)
	p = 0.0001	p = 0.001	p = 0.003
Observations	58,291	58,291	58,291
Women: All-Cause Dementia	1.14 (1.02, 1.27)	1.13 (1.01, 1.27)	1.11 (0.99, 1.24)
	p = 0.02	p = 0.03	p = 0.08
Observations	61,522	61,522	61,522

© 2022 Duchowny KA et al. *JAMA Network Open.*

eFigure 1. Example Kaplan-Meier Curves for All-Cause Dementia for the 5th Age-Decile in Men

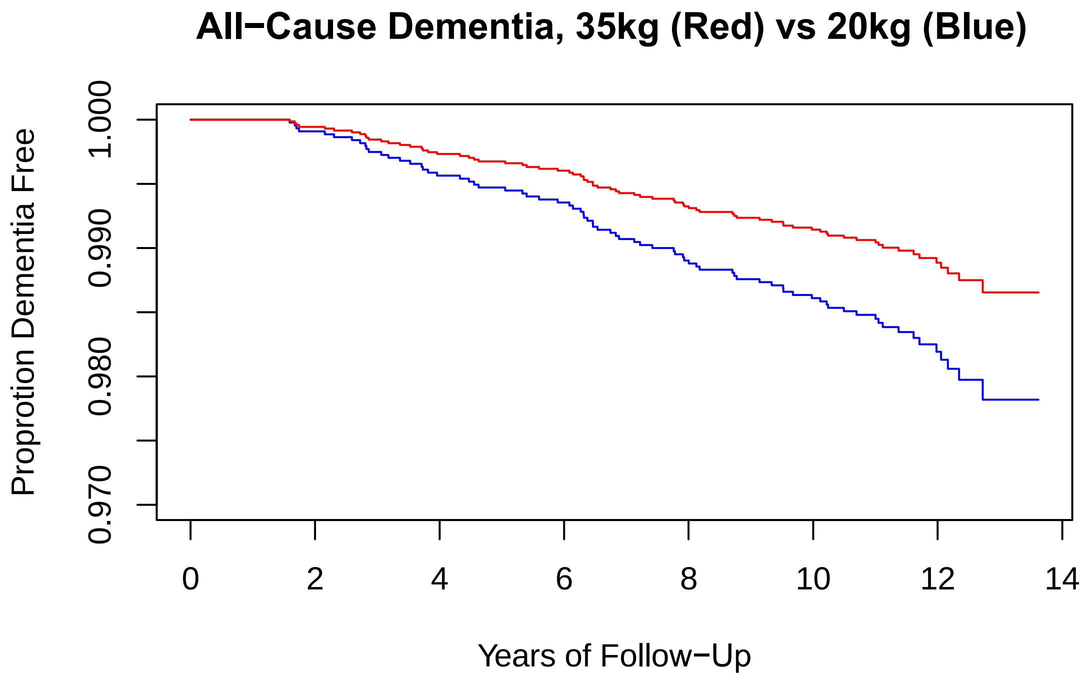

© 2022 Duchowny KA et al. *JAMA Network Open.*

eFigure 2. Example Kaplan-Meier Curves for All-Cause Dementia for the 5th Age-Decile in Women

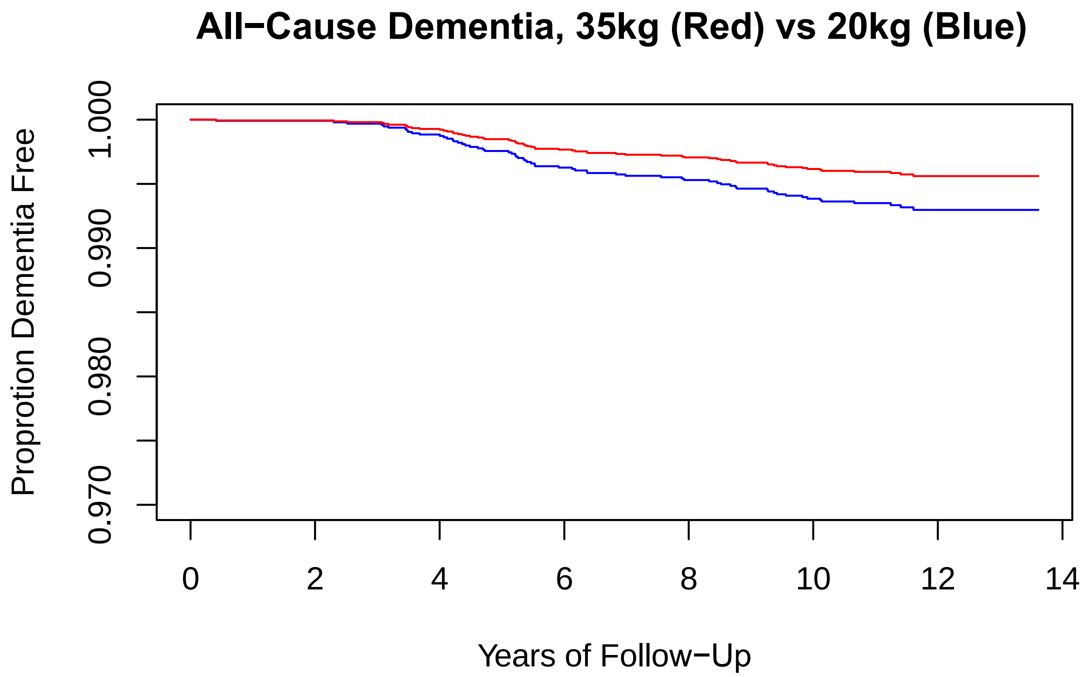

© 2022 Duchowny KA et al. *JAMA Network Open.*

eFigure 3: Reverse Causation Schematic

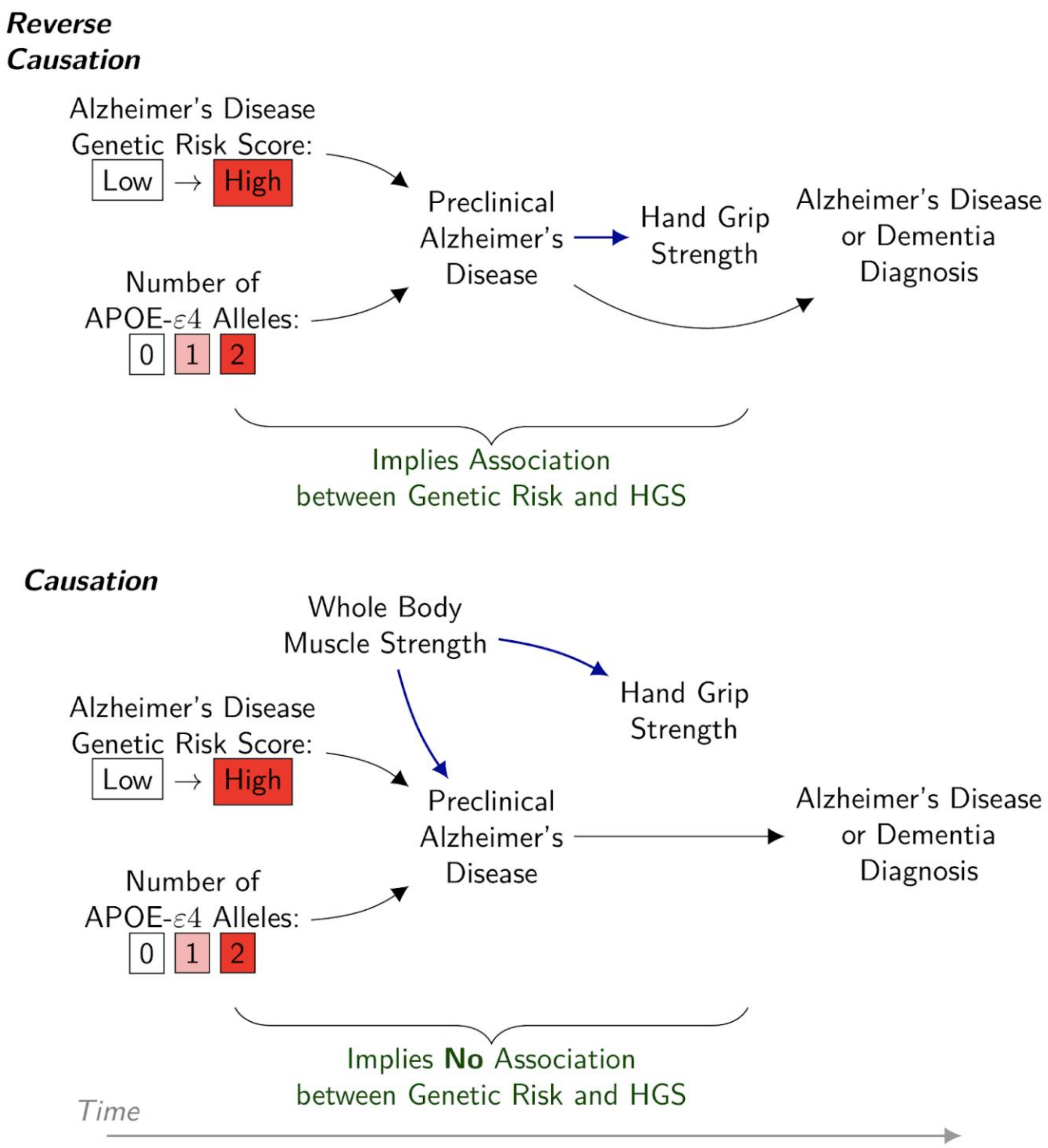

© 2022 Duchowny KA et al. *JAMA Network Open.*

eTable 16: Associations Between Polygenetic Risk Score (PGS), APOE-e4 Alleles And Handgrip Strength Stratified by Gender and Age.

	Men under 65	Women under 65	Men under 65	Women under 65	Men 65+	Women 65+	Men 65+	Women 65+
	(1)	(2)	(3)	(4)	(5)	(6)	(7)	(8)
PGS Score (+SD increase)	0.012	0.020			-0.016	-0.028		
	(-0.029, 0.054)	(-0.006, 0.046)			(-0.082, 0.050)	(-0.077, 0.020)		
	p = 0.564	p = 0.125			p = 0.638	p = 0.250		
APOE-e4 (+Allele)			-0.029	0.010			-0.019	-0.110*
			(-0.111, 0.052)	(-0.040, 0.060)			(-0.148, 0.111)	(-0.204, -0.016)
			p = 0.480	p = 0.696			p = 0.776	p = 0.022
Observations	179,994	221,349	179,994	221,349	61,376	60,369	61,376	60,369

© 2022 Duchowny KA et al. *JAMA Network Open.*
